# Design of Modified Polymer Membranes Using Machine Learning

**DOI:** 10.1021/acsami.3c18805

**Published:** 2024-04-11

**Authors:** Sarah Glass, Martin Schmidt, Petra Merten, Amira Abdul Latif, Kristina Fischer, Agnes Schulze, Pascal Friederich, Volkan Filiz

**Affiliations:** † Institute of Membrane Research, Helmholtz-Zentrum Hereon, Max-Planck-Str. 1, Geesthacht 21502, Germany; ‡ Institute of Theoretical Informatics, Karlsruhe Institute of Technology (KIT), Kaiserstr. 12, 76131 Karlsruhe, Germany; § Leibniz Institute of Surface Engineering (IOM), Permoserstr. 15, Leipzig 04318, Germany; ∥ Institute of Nanotechnology, Karlsruhe Institute of Technology (KIT), Kaiserstr. 12, 76131 Karlsruhe, Germany

**Keywords:** neural network, regression models, electron beam modification, ultrafiltration membrane, surface modification

## Abstract

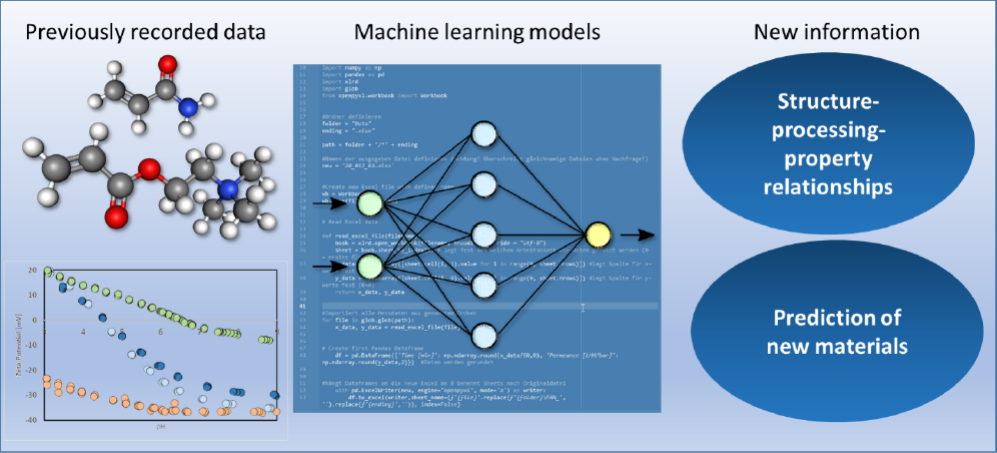

Surface modification is an attractive strategy to adjust the properties of polymer membranes. Unfortunately, predictive structure–processing–property relationships between the modification strategies and membrane performance are often unknown. One possibility to tackle this challenge is the application of data-driven methods such as machine learning. In this study, we applied machine learning methods to data sets containing the performance parameters of modified membranes. The resulting machine learning models were used to predict performance parameters, such as the pure water permeability and the zeta potential of membranes modified with new substances. The predictions had low prediction errors, which allowed us to generalize them to similar membrane modifications and processing conditions. Additionally, machine learning methods were able to identify the impact of substance properties and process parameters on the resulting membrane properties. Our results demonstrate that small data sets, as they are common in materials science, can be used as training data for predictive machine learning models. Therefore, machine learning shows great potential as a tool to expedite the development of high-performance membranes while reducing the time and costs associated with the development process at the same time.

## Introduction

Industrial, agricultural, and pharmaceutical production for human needs have created an enormous water demand and stressed water reserves. Therefore, the development of alternative water sources and improved water recycling methods is an urgent task for humanity,^1,2^ to achieve the sustainable development goals of the United Nations and to provide clean water for everyone.^3^ Membrane processes are regarded as a promising technique for the purification of water streams because they often have reasonable recovery rates and low energy demand.^4,5^ Since polymeric membranes were introduced in water treatment applications in the 1950s, their utilization can be observed for effectively eliminating bacteria, viruses, macromolecules, organic compounds, and salts from contaminated feed streams.^6^ Therefore, membrane technology is promising in several applications, not only in waste and process water treatment but also in the purification of solvents and gas separation.^1,7^ However, the membrane surface properties often limit their performance. Therefore, surface modification is an attractive strategy to customize the properties of the polymer membranes. Reducing unwanted effects such as fouling^8^ or improving the general membrane performance^9^ are common reasons to modify membranes.

The primary focus of this study was to examine the modification of polymer membranes through the introduction of positively charged amine groups. Amine-modified membranes showed great potential to adsorb and remove toxic metals^10−12^ and textile dyes^13,14^ from water. These water contaminants can cause significant harm and serious illness if consumed long term.^15^ Therefore, the complete elimination of these pollutants from water is necessary. Functionalization of membranes for those specific applications usually improves the purification performance but also influences other material and device properties. Therefore, the design of amine-modified polymer membranes is highly complex as it involves balancing multiple factors, such as the type and concentration of the amine substance, the pristine membrane, and the process parameters. Unfortunately, predictive structure–processing–property relations are barely known, making it hard to estimate these properties before experimental preparation and characterization of the membranes. Therefore, the optimization of modified membranes or the modification of membranes using new substances and strategies is often a time- and cost-intensive process.

One possibility to tackle the complexity of similar issues is the application of machine learning.^16,17^ Machine learning is a type of artificial intelligence that enables computers to learn patterns from data and subsequently make decisions based on these patterns.^18^ To do this, machine learning approaches usually use large data sets. However, in chemistry and material science, data sets are often limited to a few dozen or hundreds of data points, which leads to the fear of inaccurate predictions.^19^ Nevertheless, machine learning showed excellent predictive capability in this field as well.^20^

One membrane application for which the machine-learning models were used in the past is the preparation of organic solvent nanofiltration (OSN) and reverse osmosis (OSRO) membranes. For example, Ignacz et al.^21^ used graph neural networks to identify the most critical solvent parameters affecting the rejection of solutes by polyimide OSN membranes. Other works focused on the optimization of operation conditions,^22^ performance parameters such as permeance and rejection,^23−25^ and solvent–membrane interactions.^26^ When validated with experimental data, the applied machine-learning models often showed excellent accuracy and predictive capability.^17,27^

In this study, we applied machine learning methods to data sets containing performance parameters of electron beam modified membranes. This method can be used to graft organic molecules on membranes to improve their performance.^28−30^ The aim was to prepare membranes with a positive surface charge and a highly pure water permeance at the same time. Herein, we used previously recorded data sets to explore relationships among the chemical structure of the modification substance, the parameters used in the modification process, and the resulting properties of the modified membranes. Additionally, we used the data-driven approach to predict the properties of membranes modified with new modification substances. In this study, we showed that instead of starting an independent and time-consuming optimization process, machine learning can be a tool to predict the properties of a modified membrane quickly and accurately before preparation. Thus, selecting promising membranes with high expected performance before preparation is a promising approach to save time and resources.

## Experimental Section

### Materials

All acrylic amine compounds (*N*-[3-(dimethylamino)-propyl]-methacrylic amide (DMAPMA), methacrylic acid-2-(dimethylamino)-ethyl ester (DMAEMA), 2-trimethylammoniumethyl methacrylate chloride, [2-(acryloyloxy)ethyl]trimethylammonium chloride, (3-acrylamidopropyl)trimethylammonium chloride, 3-(methacryloylamino) propyl-trimethylammonium chloride, 2-(dimethylamino)ethyl acrylate, 2-aminoethylmethacrylamide hydrochloride, acrylamide, and methacrylamide), and dimethylformamide (DMF) were purchased from Sigma-Aldrich (St. Louis, MO, USA). γ-Butyrolactone (GBL) was obtained from Merck KGaA (Darmstadt, Germany). Polyacrylonitrile (PAN) powder (>99% acrylonitrile, homopolymer, *M*_w_ = 200 000 g/mol) was obtained from DOLAN (Kelheim, Germany). All chemicals were used without further purification.

### Membrane Preparation

Two different PAN membranes were prepared from a procedure previously described by Scharnagl et al.^31^ In brief, PAN powder (8 or 10 wt %, respectively) was dissolved in dimethylformamide (DMF) and γ-butyrolactone (GBL). Afterward, the solution was coated onto a nonwoven support using a doctor blade with a gap height of 200 μm. The membranes were drop-casted in tap water at room temperature, washed with water, and dried.

The membranes were named “M1” (prepared from 8 wt % PAN solution) and “M2” (prepared from 10 wt % PAN solution). The pristine membranes were characterized thoroughly as described in the Supporting Information. Retention analysis and SEM images are shown in Figures S1, and S2 (Supporting Information page 3). A brief overview of the characteristics of the pristine membranes is shown in Table 1.

**Table 1:** Characterization of Pristine Membranes M1 and M2

	M1	M2
water contact angle(deg)	52.6 ± 1.8	45.1 ± 1.7
surface pore size(nm)	11.0 ± 6.6	9.5 ± 4.5
surface porosity(%)	9.5 ± 0.6	4.7 ± 0.4
pure water permeance (LMH/bar)	2008 ± 142	1182 ± 39
molecular weight cutoff(kDa)	600	504
surface roughness*R*_a_ (nm)	9.29 ± 2.81	5.89 ± 0.76

### Membrane Modification

The membranes—M1 and M2—were coated with amine-containing polymers using electron-beam modification. The modification substances used were either acrylates or acrylamides containing an amino group. Thereby, solutions (250 mL each) containing 0.5, 1.0, 2.5, 5.0, 10.0, 15.0, or 20.0% (w/v) of the respective modification substances were prepared. The 15 × 20 cm^2^ membrane pieces were immersed in the respective solution for 15 min. Afterward, the membranes were transferred to a roll-to-roll electron beam irradiation system.^32^ The wet, immersed membranes were exposed to electron beam irradiation using either a dose of 150 or 200 kGy (160 keV acceleration voltage, 2 m/min conveyor speed). No significant increase in the temperature was detected during the irradiation process. After irradiation, the membranes were washed in deionized water twice for 30 min each and dried at room temperature.

A complete list of the prepared membranes is displayed in Table S1 (Supporting Information pages 4–7).

### Membrane Characterization

The modified membranes were characterized by measuring the pure water permeance (PWP) and zeta potential of the membranes.

The PWP was measured in dead-end mode using an inbuilt device. It was measured using a circular membrane piece with a diameter of 2.0 cm corresponding to an active area (*A*) of 1.68 cm^2^. Ultrapure water was used. The permeance (*P*) was calculated using the following equation:
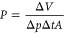
1

where Δ*V* is the difference in volume, Δ*p* is the transmembrane pressure (here usually, 2 bar), Δ*t* represents the time interval (1 min), and *A* is the membrane area. The permeance is given in LMH/bar (L/(m^2^ h bar)). From the resulting time-dependent permeance curve, the permeance value after 5 min of measurement was used in this study.

The zeta potential was measured using a SurPASS Eco 3 instrument from Anton Paar (Graz, Austria). The streaming potential method was used. The electrolyte solution used was a 0.01 M NaCl solution. The pH was adjusted by using 0.05 M NaOH and 0.05 M HCl. All solutions were prepared by using ultrapure water. The membranes were rinsed with the electrolyte solution, until the membrane was completely swollen. The measurements were performed in the pH range from 3 to pH 9. At each pH, the zeta potential was measured four times.

### Data Preparation and Feature Selection

The concentration of the modification solution (continuous values between 0.5 and 20.0), the used membrane (binary value, i.e., M1 was assigned 1 and M2 was assigned 0), and the dose of the electron beam irradiation (categorical value, 150 or 200) were used as features to describe the preparation process. Additionally, the modification substance was described using the p*K*_a_ value of the amine group (continuous values between 3.8 and 14.0). For the calculation of the p*K*_a_ values, the software MolGpKa was used.^33^ Quaternary amine groups were assigned a p*K*_a_ value of 14. Additionally, the modification substances’ acrylic functional groups were described by two features. The first feature (binary) described whether the substance is an acrylate (hence, an acrylamide). The second feature (also binary) described whether the acrylic group was methylated (1) or not (0). A complete list of the substances with the assigned features is given in Table S2 (Supporting Information page 8).

The zeta potential and pure water permeance (PWP) values of the modified membranes were used as labels for the machine-learning-based material design approach. As common in experimentally collected data sets, the number of samples per characterization method varied. 52 membranes were used for the PWP predictions and 42 for the zeta potential predictions. In the case of the PWP, each membrane corresponded to one data point containing 6 features (concentration, p*K*_a_, dose, acryl group, methyl group and membrane) and one output value (PWP after 5 min). Since the zeta potential is pH dependent, the pH value was used as an additional feature in this case. This feature was necessary to enable the prediction of the full zeta potential curves. Each zeta potential curve of a membrane contained about 60 measurements. Therefore, the data set of the zeta potential contained 2144 data points but only 42 independent membrane samples. Seven features were used in each data point (concentration, p*K*_a_, dose, acryl group, methyl group, membrane, and pH) and one output value (zeta potential).

### Application of Regression Models

As a baseline, regression models from the Scikit-learn library were trained by using the gathered data. The first step was to import the respective data sets. The data sets are available in Glass et al.^34^ Afterward, the input features and labels (zeta potential or permeance, respectively) were split into a training and a test subset. 15% of the data were randomly placed in the test data set, and 85% were randomly placed in the training data set. The data were scaled using the sklearn StandardScaler. Five regressors (random forest, gradient boosting, Ada boost, extra tree, and linear regression) from Scikit-learn were used to fit the data. After training on the training set, the features of the test data set were used to predict the output values (PWP or zeta potential, respectively) using the fitted regressors. The feature importance was calculated for all regressors except for the linear regression to plot the effect of each feature on the output values. The mean absolute errors and coefficients of determination (*R*^2^) of the predicted output values compared with the output test data) were calculated. Additionally, the standard deviation of the labels of the output test subset was calculated.

The gradient boost regressor showed the highest determination coefficient for both data sets. Therefore, this regressor was used for further validation. A leave-one-out cross-validation approach was used. In this validation method, one sample of the data set was used as the validation set and was predicted using all of the other values as training data for the regression. This was repeated for all samples, and the predicted values were compared with the measured ones.

### Application of a Neural Network

In the second step, the data were used to train a neural network using Keras library.^35^ The data sets were imported, and the features and labels were scaled using the Scikit-learn StandardScaler. The data were used to train a neural network containing two hidden layers. The first layer contained 64 neurons; the second layer contained 16 neurons. Both layers were fully connected layers. The model was fitted, and the SHAP values for all features were calculated.^36^

To evaluate the neural network, two new data sets were created, excluding 3 representative samples of each original data set.^34^ The excluded samples were:

– 1% acrylamide on M1 prepared at 200 kGy

– 5% methacrylamide on M1 prepared at 200 kGy

– 2.5% ammoniumethyl acrylate on M2 prepared at 200 kGy

The model was trained using the entire new training data set. 100 independent models with different weight initializations were trained. With each model, the zeta potential (depending on pH) or the PWP, respectively, of all three excluded samples were predicted. The averages and standard deviations of the 100 predictions were calculated and plotted against the experimentally measured values.

The training curves (of each training loop) for the training and validation data are plotted in Figure S4 (Supporting Information page 9).

### Prediction of New Modified Membranes Using the Neural Network

Finally, the averages of the trained neural networks were used to predict the PWP and zeta potentials of membranes modified with new modification substances. Thus, two new substances with properties similar to those of the previously used modification substances were used. The substances were *N*-[3-(dimethylamino)-propyl]-methacrylic amide (DMAPMA) and methacrylic acid-2-(dimethylamino)-ethyl ester (DMAEMA). The zeta potentials and the PWPs of both membranes modified with the two substances were predicted depending on the irradiation dose and the concentration. Zeta potential values and PWPs of all 120 membranes using either DMAEMA on membrane M1 or DMAPMA on M2 were predicted for modifications at different irradiation doses and with different concentrations. The same range of dose and concentration values as those in the training data was used.

The predictions were experimentally validated by preparing a total of eight new membranes. Four membranes were prepared by modifying M1 with DMAEMA, and the other four membranes were prepared by modifying M2 with DMAPMA. The membranes were chosen using application-relevant criteria. The first criterion was that the membranes should have a high PWP (≥550 LMH/bar). The second criterion was that the membranes should have a high zeta potential (at a neutral pH). We have chosen membranes with a) the highest zeta potential predicted by the neural network, b) a positive zeta potential using the lowest concentration, and c) the highest zeta potential using a dose of 200 kGy. Additionally, one membrane at a random concentration and dose value within the predicted range was prepared. After preparation, the membranes were analyzed, and the predicted and measured values were compared.

## Results and Discussion

### Regression Model

**Figure 1:**
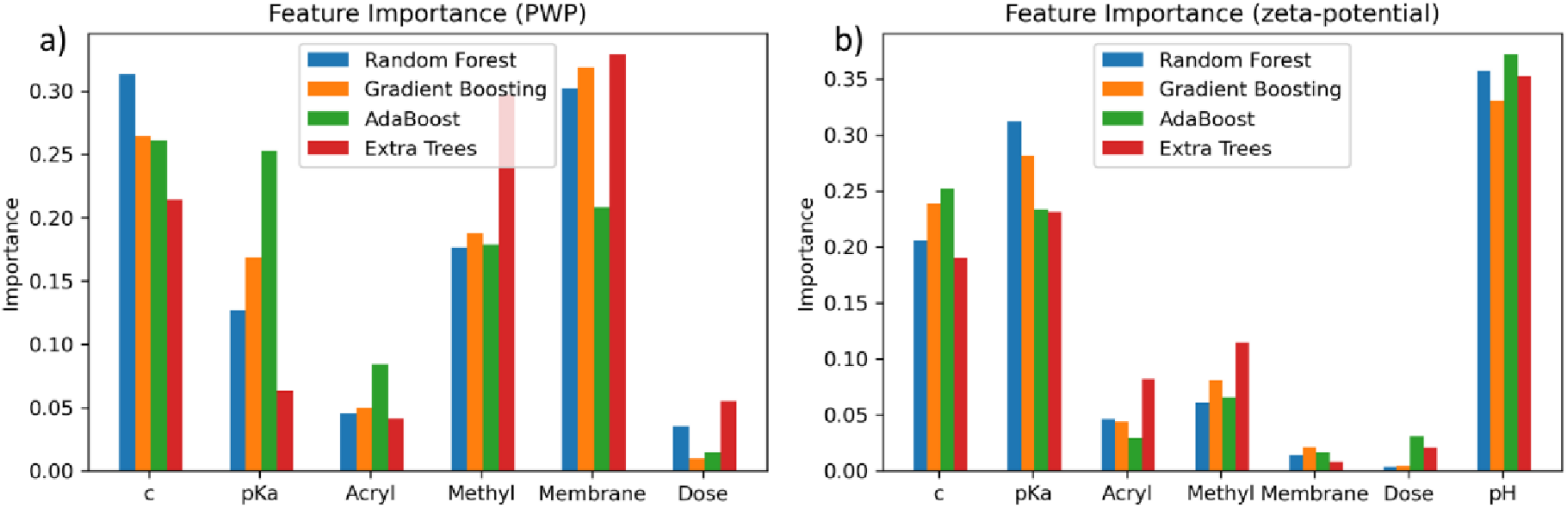
Importance of the chosen features on the regression of the (a) PWP and (b) zeta potentials shown for four different regressors.

In general, structure–processing–property relationships are essential for the development of modification strategies. In the first part of this study, we used different regression models to analyze the relationships between the properties of modified membranes with the structure of the modification substances and the parameters used in the modification process, such as the electron beam irradiation dose (“Dose”) or applied concentration (“c”). In Figure 1, the feature importance of the respective features for four regression models is displayed. It was used to identify the features with the most influence on both properties—PWP and zeta potential. The importance of each feature was affected by the model used. However, the feature importance showed a trend similar to that for all four used models.

The PWP (Figure 1a) was mainly affected by the concentration of the modification substance (“c”) and the pristine membrane (“Membrane”). These two features’ importance was highest and above 0.20 for all four regressors. This means they had the highest impact on the PWP of the modified membranes. Additionally, the methyl feature showed high importance (above 0.15). Therefore, methylated compounds led to membranes with a different PWP compared to unmethylated modification substances. The electron beam irradiation dose (“Dose”) and the acryl functionality of the modification substance (“Acryl”) had low importance (<0.10) and, therefore, a low impact on the PWP of the modified membranes. The zeta potential, on the other hand (Figure 1b), was mainly impacted by the pH value of the measurement. While not a parameter of the membrane modification, the pH value naturally affects the zeta potential. In general, a decrease in the pH value leads to an increase in the zeta potential because of the increase in the proton concentration. The feature with the second highest importance was the p*K*_a_ value (>0.20). Therefore, the p*K*_a_ value of the amine group had the highest impact on the resulting zeta potential. Another feature with high importance was the concentration (>0.15) of the modification substance. All other features had either a low impact (importance of the acryl and methyl group <0.10), or almost no impact on the zeta potential (importance of the membrane and dose <0.05).

These results showed that the chemical structure of the modification substances affected the resulting properties of the membrane. Whether the structure contained, for example, an acrylic group or an acrylamide group was important for the PWP of the modified membranes, in particular. However, a features’ importance of the PWP or the zeta potential was not necessarily linked to a chemical or physical phenomenon that affected the properties. It represents a statistical relation between the input and output values. Therefore, concluding any physicochemical mechanisms from these data was not feasible. Nevertheless, the importance of the features can help to understand which of the chosen features and parameters likely affected the properties of the modified membranes. Therefore, new modification substances with specific chemical structures and functional groups or new modification strategies can be chosen based on these data.

To evaluate the quality of the regression models, we calculated the coefficients of determination (*R*^2^), and the mean absolute errors of the regressors were calculated. The respective values are displayed in Table 2. In general, the coefficient of determination is high if a model replicates the outcome well. In this study, all regressors (except the linear regressor for the zeta potential) showed high determination coefficients of 0.7 or higher, reaching 0.96 for the gradient-boosting regression model. The *R*^2^ values were high compared to other studies using small data sets, where ensemble methods often showed determination coefficients between 0.5 and 0.9.^37−39^ This showed that regression models were capable of replicating the output values. The standard deviation of the PWP test data set was 763.2 LMH/bar. The mean absolute errors of all of the regressors were at least 50% smaller. The gradient boosting model showed a mean absolute error of 122.0 LMH/bar, which was significantly lower than the other values. Similar results were obtained for the zeta potential. The standard deviation of the test set was 16.1 mV. The gradient boosting model again showed the lowest mean absolute error (2.1 mV) and highest *R*^2^ (0.967). The mean absolute errors of the gradient boosting model for both—PWP and zeta potential—were in the same range as the typical mean absolute error of the respective measurements. As seen in previous studies before,^39^ the tree-based ensemble methods showed good prediction potential even for small data sets.

**Table 2:** Coefficient of Determination (*R*^2^) and Mean Absolute Error of the Training Data for the PWP and the Zeta Potential Using the Five Regressors

regressor	*R*^2^ (PWP)	mean absolute error (PWP)(LMH/bar)	*R*^2^ (zeta potential)	mean absolute error (zeta potential) (mV)
random forest	0.820	289.3	0.943	2.8
gradient boost	0.963	122.0	0.967	2.1
ada boost	0.863	250.1	0.784	6.5
extra tree	0.728	351.4	0.899	3.7
linear regression	0.749	310.7	0.550	8.7

**Figure 2:**
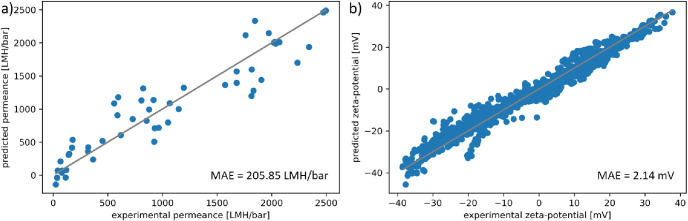
Experimental values compared to the values predicted by the gradient boosting model using the leave-one-out cross-validation for (a) PWP and (b) the zeta potential. The gray line displays a linear plot with a slope of 1, representing an ideal prediction. Mean absolute error (MAE) is shown in the respective lower right corner.

The gradient boosting regressor showed the highest determination coefficient and the lowest mean absolute error for both output values. Therefore, the leave-one-out cross-validation was used to analyze the quality of the model further. The results are displayed in Figure 2. The predicted values of PWP and zeta potential were in good accordance with the respective experimental values, and there were no extreme outliers in the prediction. The training performance is shown in Figure S3 (Supporting Information page 9).

This showed that the collected data were usable for machine learning approaches, even though the amount of data was relatively small. Additionally, it showed that the features were chosen well and were suitable for describing the output values. At the same time, it is important to note that the zeta-potential prediction, specifically in the leave-one-out cross-validation case, is a pure interpolation task, as there are always training data points that are “close” to the test data points. Since the first results using regression models were very promising, the application of neural networks as another machine learning tool was evaluated. Therefore, the collected data were applied to the training of a neural network.

### Neural Network

Regression models are, in general, intuitive, interpretable, and effective for capturing nonlinear relationships within data. Tree-based models, such as decision trees, random forests, and gradient boosting machines, make predictions by recursively partitioning the input space into regions and assigning a constant value to each region. Regression models are widely used in a variety of fields.^40^ While they are perfectly suitable for small- to medium-sized data sets due to their simplicity and interpretability, neural networks excel in handling complex, large-scale, and high-dimensional data. Neural networks, inspired by the structure of the human brain, consist of interconnected layers of artificial neurons. Neural networks are capable of learning complex hierarchical representations of data, enabling them to capture intricate patterns and relationships. Therefore, they may be better suited for generalization of the data and predictive purposes.^41^

Similar to the regression models, the neural network was evaluated by calculation of the coefficients of determination (*R*^2^) and the mean absolute errors. In both cases, the neural network was slightly superior to the regression models. The coefficients of determination (0.968 for PWP and 0.984 for zeta potential, respectively) were marginally higher compared to the ones of the regression models (Table 2), and the mean absolute errors were lower (105.9 LHM/bar for the PWP and 1.6 mV for the zeta potential).

**Figure 3:**
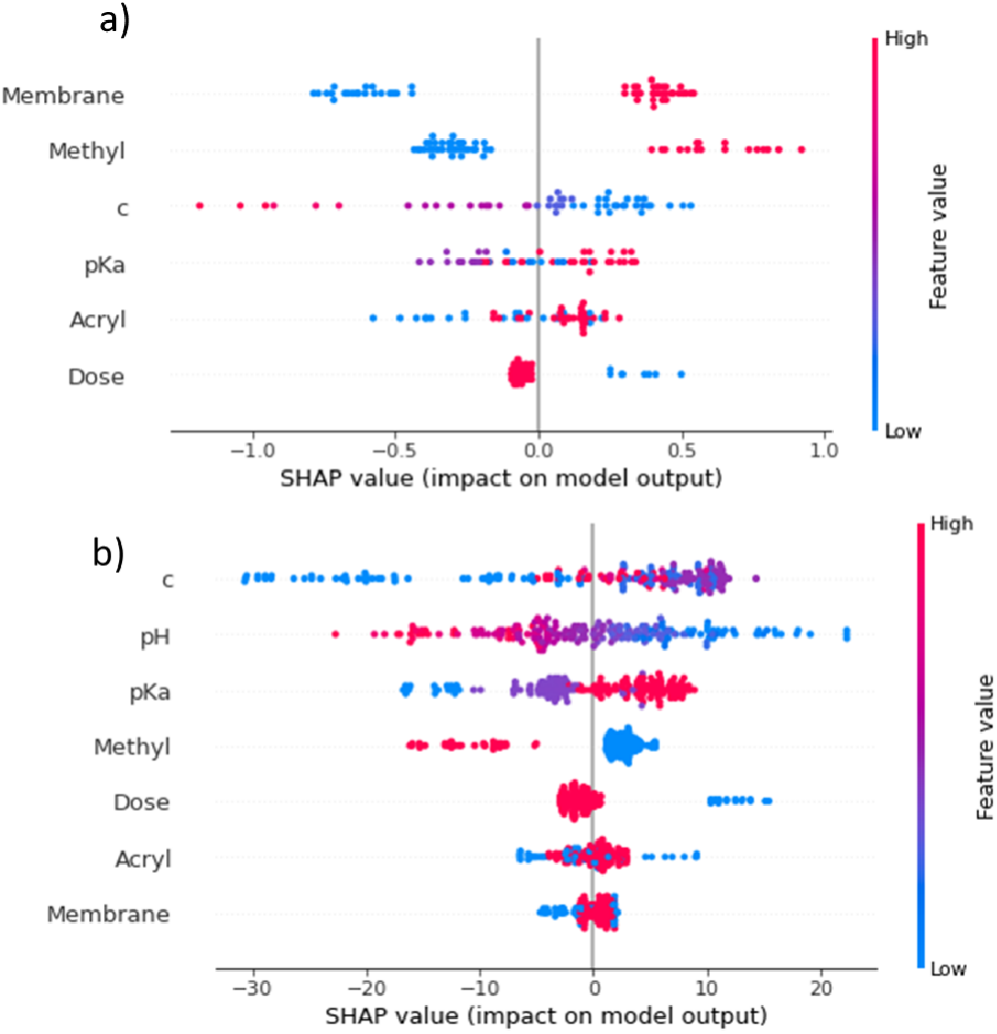
Impact on the model output displayed using Shapley values of all features for (a) PWP and (b) zeta potential. Feature values are displayed using a color gradient and sorted from highest to smallest impact. With pink representing the highest value, purple intermediate values (if applicable) and blue the lowest. Features were sorted by impact from top to bottom.

To better understand the predictions made by the neural networks, the Shapley values of the features were calculated (Figure 3). Shapley values are a method to display a feature’s contribution to the output values.

Figure 3a shows the effect of the features on the PWP by using the neural network. The results showed a similar trend, as displayed in Figure 1a. Again, the membrane, the methyl feature, and the concentration had the highest impact. However, the Shapley values allowed for a more detailed insight. They showed whether the output values were high or low depending on the values of the respective feature value.

As seen in Figure 3a, the PWP was high for membrane M1 (feature value of 1; shown in pink) and for the modification substance that was methylated (methylated substances had a value of 1; shown in pink). On the contrary, it was low for membrane M2 (feature value = 0; shown in blue). Additionally, the higher the concentration of the modification substance, the lower was the PWP. The p*K*_a_ value and the acryl feature had a lower and less unambiguous impact on the model’s output. The irradiation dose finally had a minor impact. However, low dose values (150 kGy) led to slightly higher PWP compared with high values (200 kGy).

Figure 3b displays the impact of the feature values on the zeta potential. The concentration and the p*K*_a_ value had the highest impact. In both cases, high values led to high zeta potential. Again, the impact of the pH value was not considered since it was not a process parameter. The other features had only a minor impact on the zeta potential. However, the presence of the methyl or amide feature in the modification substance led to slightly lower zeta potential values.

The Shapley values helped us to interpret the predictions made by the neural networks. Additionally, they can help in choosing new modification substances in the future. For example, in this study, choosing an acrylic compound instead of an acrylamide can be beneficial since membranes modified with substances having an acrylic functional group had a slightly higher zeta potential as well as high PWPs. Additionally, choosing a substance with a high p*K*_a_ value can be of advantage. Membranes that were modified with those substances had a high zeta potential, while PWP was not affected significantly.

**Figure 4:**
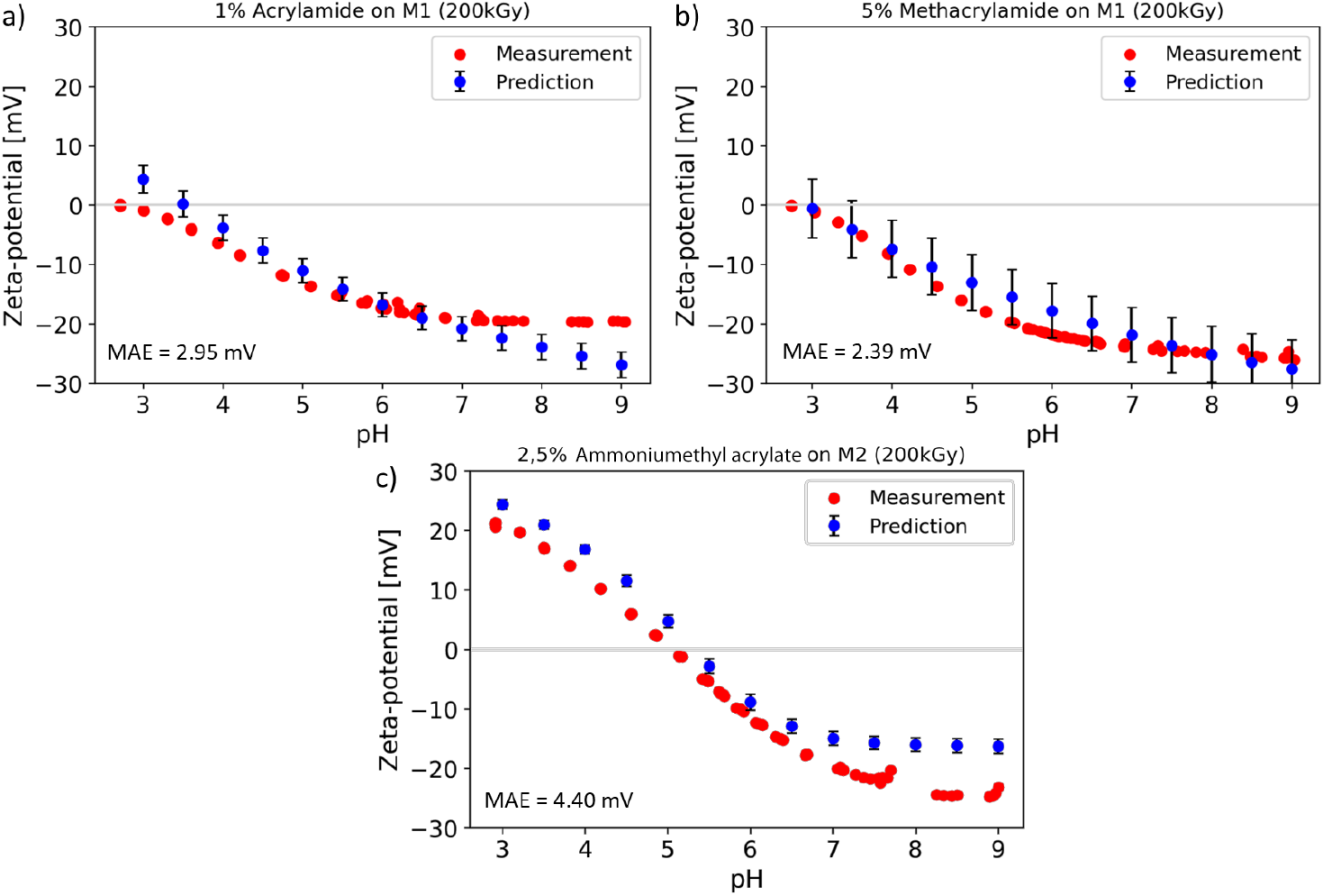
Zeta potential curves predicted by neural network compared to the experimentally measured zeta potential curves for three known modification substances in new concentrations (a) acrylamide on M1 (1 wt %), (b) metharcylamide on M1 (5 wt %), and (c) ammoniumethyl acrylate on M2 (2.5 wt %). All modifications were predicted and prepared at 200 kGy. Red dots display the measurement, and blue dots display the mean predicted value. The displayed predicted values were averages of predictions by 100 independently trained neural networks. Error bars (black) display the standard deviation of the predictions (*n* = 100). Mean absolute error (MAE) is shown in the respective lower left corner.

**Figure 5:**
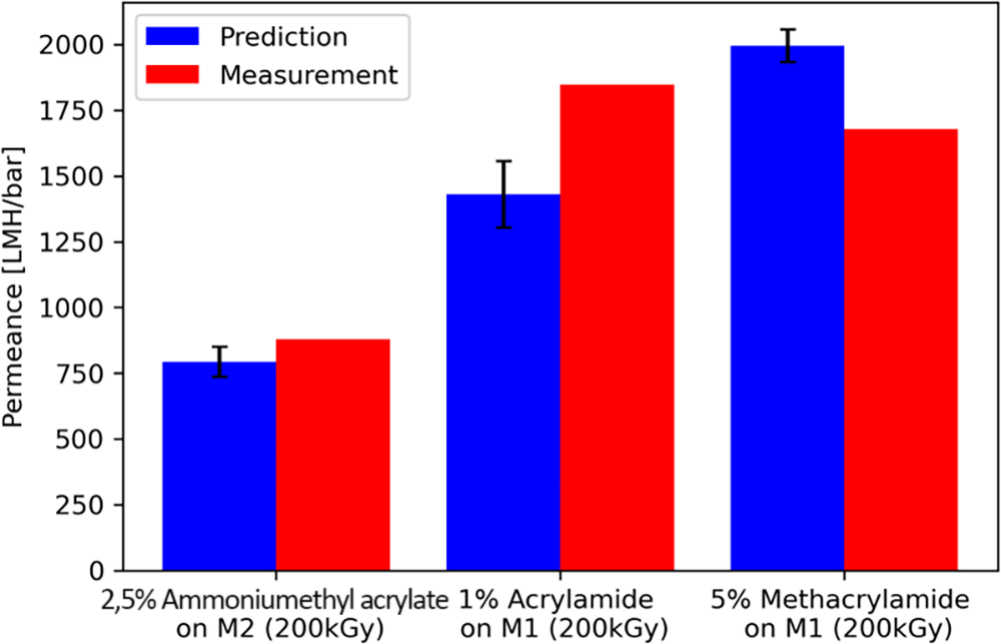
PWP predicted by neural network (blue) compared to experimentally measured PWP (red) for three known modification substances in new concentrations. The displayed predicted values were averages of 100 predictions. Error bars (black) display the standard deviation of all predictions (*n* = 100). The mean absolute error of the predictions was 273.9 LMH/bar.

For further validation, three representative membranes’ data were erased from the data sets and the PWP as well as the zeta potential were predicted using the neural network. The predicted zeta potentials compared with the predicted values are presented in Figure 4. The modification substances used for the predictions were also present in the training data. Therefore, the predicted zeta potentials were in good accordance with the measurements for all three examples. The standard deviations by the 100 independently initialized neural networks were slightly underestimating model errors, which could be improved through bootstrapping or uncertainty calibration methods. However, the shape of the measurement curve, the isoelectric point (IEP, pH where the zeta potential is zero), and the magnitude of the zeta potential were all represented well by the prediction. Additionally, in Figure 5, the predicted PWPs were compared with the actual measured PWPs. Again, the predicted PWPs were in good accordance with the measured values. This showed that the neural network was well-suitable for predictive purposes.

### Prediction of New Modified Membranes Using the Neural Network

Besides understanding structure–processing–property relationships, predicting the properties of new and unknown materials is the most interesting application for machine learning approaches in material science. Therefore, modification of the membranes with two new substances was predicted using the neural network in this study. The two substances chosen were DMAEMA and DMAPMA because they were similar to the modification substances of the previously recorded data sets but not part of them. Concentration, doses and p*K*_a_ values of the chosen substances were in the same range as in the training data. This means that no extrapolation of the feature values was performed. In total, 120 modified membranes using DMAEMA on membrane M1 and 120 modified membranes using DMAPMA on membrane M2 were predicted. Preparing and analyzing all of these membranes would require several months of work and substantial amounts of material and equipment time. However, computing the neural network for the prediction of the zeta potential and PWP was possible in less than 1 h. This showed the huge time-saving ability of employing machine learning approaches.

**Figure 6:**
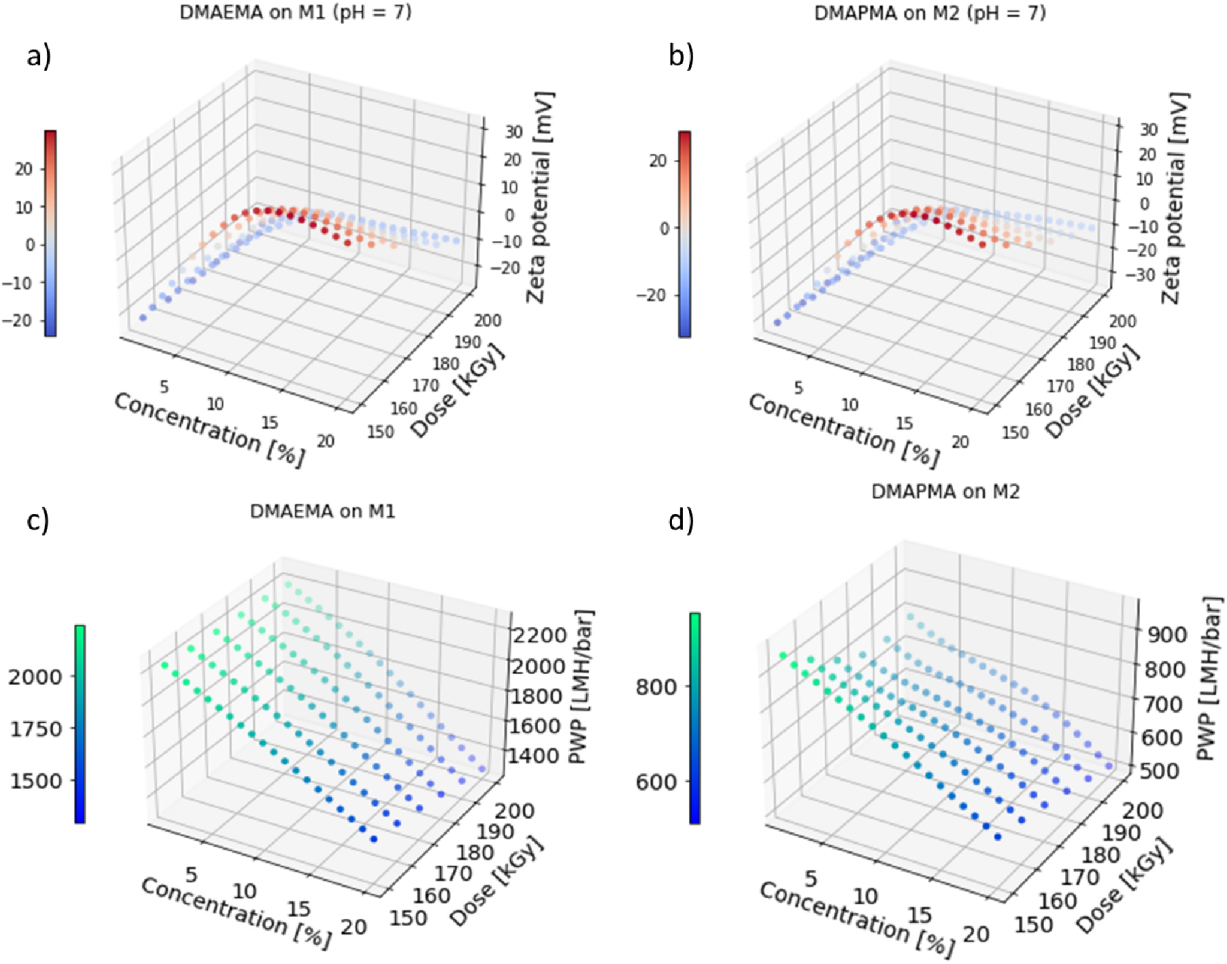
Prediction of zeta potential at pH = 7 (a and b) and PWP (c and d) for membranes modified with two new substances depending on the applied dose and concentration. The zeta potential and PWP were predicted either for DMAEMA on membrane M1 (a and c) or for DMAPMA on M2 (b and d). The zeta potential values are displayed using a color gradient from red (positive values) to blue (negative values). The PWPs are displayed using a color gradient from green (high values) to blue (low values). The displayed predicted values were averages of 100 predictions.

The zeta potentials (at pH = 7) and PWPs of the newly predicted membranes are displayed in Figure 6. The predicted zeta potentials (Figure 6a,b) were in both cases notably higher at high concentrations and low irradiation doses. Other studies using amine substances showed an increase in zeta potential after electron-beam modification of the membranes as well.^42−44^ Breite et al. showed an increase in zeta potential from −43 to +34 mV modifying a polyether sulfone membrane using tetraethylpentamine at a dose of 200 kGy. Therefore, the trend predicted by the model was in accordance with former studies.

In the case of DMAEMA on M1 (Figure 6a), the lowest concentration with a positive zeta potential was 6% modification of the substance at an irradiation dose of 150 kGy. At irradiation, doses of 190 kGy and above, no positive zeta potential were predicated at all. The highest zeta potential was predicted for a membrane prepared using 14% modification substance at 150 kGy.

In the case of DMAPMA on M2, the highest zeta potential was predicted for a membrane modified with 14% DMAPMA at 150 kGy. 6% modification substance (at 150 kGy) was the lowest concentration necessary to prepare a membrane with a positive zeta potential. Again, no membrane modified with 190 kGy or more was predicted to gain positive zeta potentials.

The PWPs of the newly predicted membranes (Figure 6c,d) decreased with increasing concentration of the modification substance and irradiation dose in both cases. The general trend was seen in former studies using acrylic compounds in electron-beam treatment of membranes as well.^45−47^ For example, Xu et al.^47^ presented a decrease in PWP from 7.7 LMH/bar to 4.7 LMH/bar when increasing the acrylate concentration from 5 wt % to 15 wt %. However, it should be noted that a direct comparison to former works in which membranes were modified was not possible due to varying performance and materials of the original membranes and different modification regimes. Therefore, further studies need to be conducted to analyze a greater variety of membranes and modification methods.

**Figure 7:**
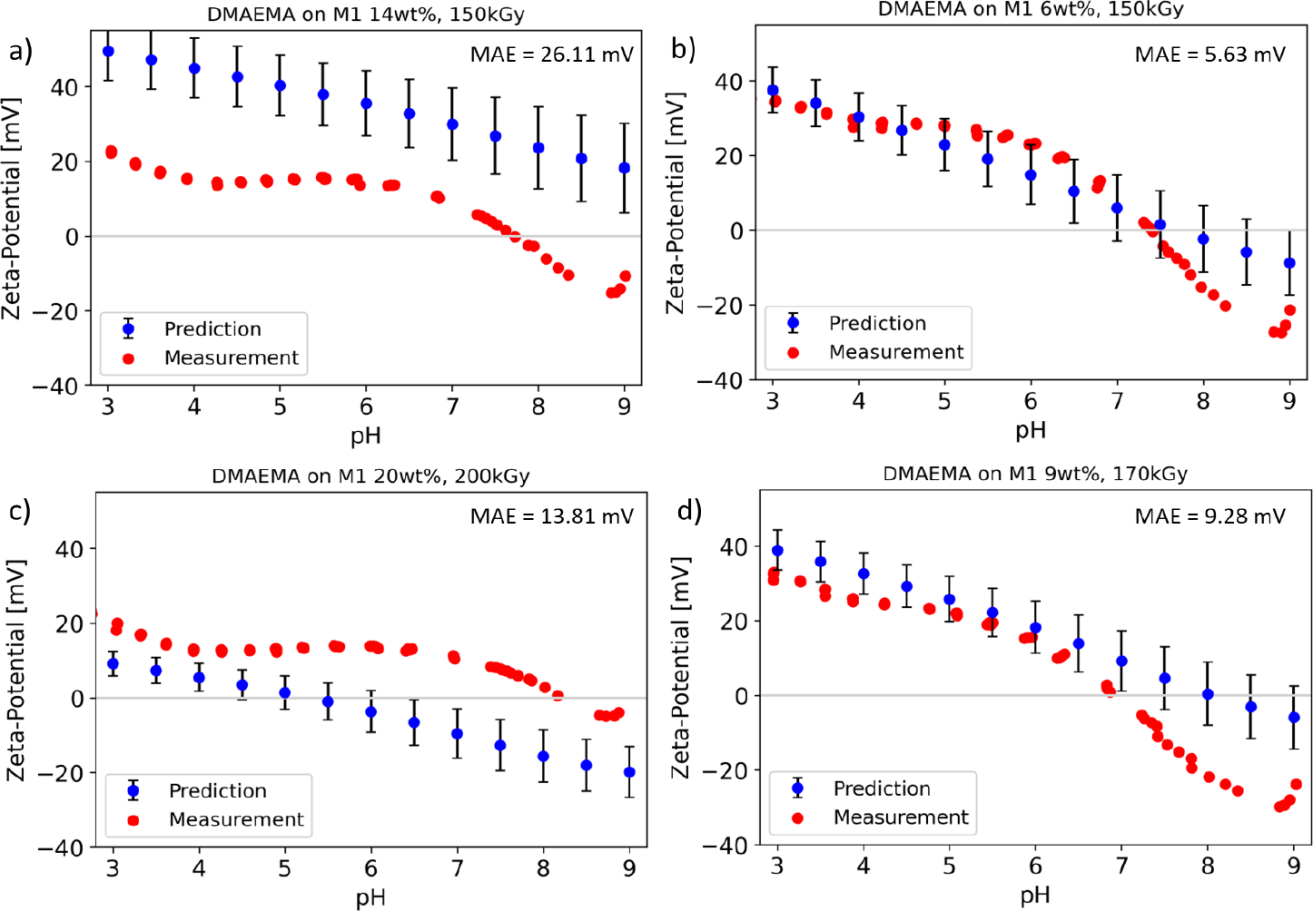
Measured zeta potential curves (red) of the M1 membrane modified with a) 14 wt % DMAEMA at 150 kGy, b) 6 wt % DMAEMA at 150kGy, c) 20 wt % DMAEMA at 200 kGy, and d) 9 wt % DMAEMA at 170 kGy compared to the predicted curves (blue). The displayed predicted values were averages of 100 predictions. Error bars (black) display the standard deviation of all predictions (*n* = 100). Mean absolute error (MAE) is shown in the top right corner.

**Figure 8:**
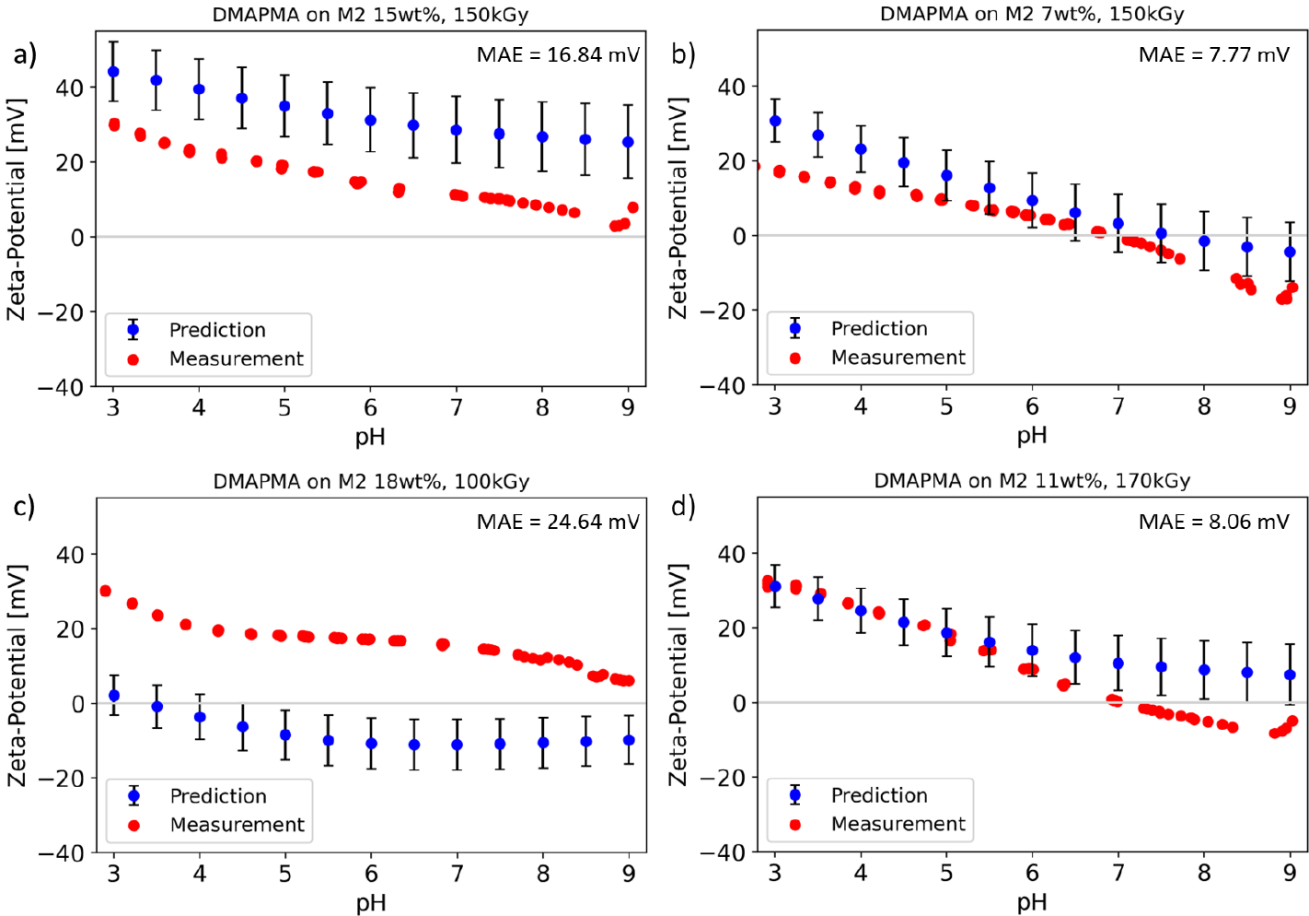
Measured zeta potential curves (red) of the M2 membrane modified with a) 15 wt % DMAPMA at 150 kGy, b) 7 wt % DMAPMA at 150kGy, c) 18 wt % DMAPMA at 200 kGy, and d) 11 wt % DMAPMA at 170 kGy compared to the predicted curves (blue). The displayed predicted values were averages of 100 predictions. Error bars (black) display the standard deviation of all predictions (*n* = 100). Mean absolute error (MAE) is shown in the respective upper right corner.

**Figure 9:**
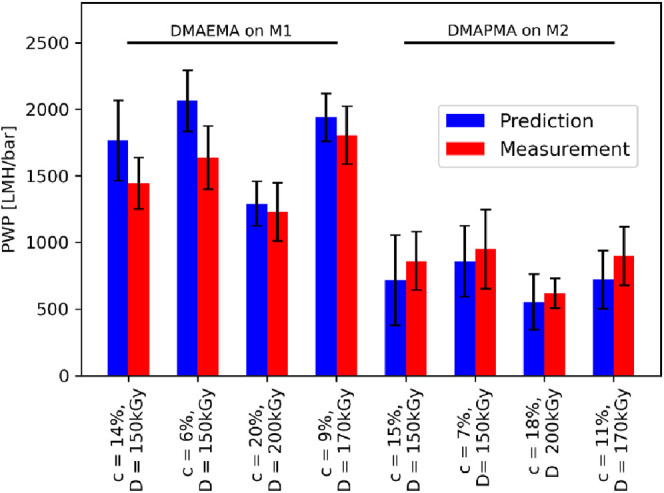
Measured PWPs (red) compared to values predicted by the neural network (blue) for the M1 membrane modified with DMAEMA (left) and the M2 membrane modified with DMAPMA (right). Error bars (black) display the standard deviation of all predictions (*n* = 100) and measurements (*n* = 3). The mean absolute error of the predictions was 177.5 LMH/bar.

The membranes using DMAPMA on M2 for the modification had significantly lower predicted PWPs (500–950 LMH/bar) compared with the one using DMAEMA on M1 (1300–2200 LMH/bar). Using these predictions, application-relevant modified membranes were identified, and each of four membranes using both new modification substances were prepared. The zeta potentials and the PWPs of the newly modified membranes were measured and compared to the predictions. The results were displayed in Figures 7– 9.

In Figure 7 the comparison between the predicted and measured zeta potential curves of DMAEMA on membrane M1 are displayed. The predictions were in good agreement with the measured values for low and intermediate concentrations of the modification substance (Figure 7b,d). The mean absolute error of these predictions was low (below 10 mV). The magnitude of the zeta potential and the IEP were predicted very well. In the alkaline pH range, the prediction was slightly lower than the measurement. Additionally, the predicted curves were smoother compared to the actual measurement because of the averaging of the predictions. This did not precisely reflect the behavior of the measurements precisely. However, the predictions were still close to the measured values and predicted the trends of the curves.

Figure 7a shows the zeta potential curves of the modified membrane with the highest predicted zeta potential. In this case, the predicted curve was significantly higher than the measured one. This can be explained by the relatively small data set and the limited number of membranes prepared with concentrations above 10% within the training data. A similar behavior can be seen in Figure 7c. The membrane used in this example was prepared using 20% of the modification substance, which represented the edge of the training data. Herein, the predicted data were slightly lower than the measured zeta potential curves. However, considering the small amount of data and the limited number of training data for high concentrations, all predictions were satisfactory.

Figure 8 shows the predicted and measured zeta potential curves for the second set of modified membranes—DMAPMA on membrane M2. Similar trends, as in Figure 7, were observed. The two membranes prepared at intermedium concentrations (Figure 8b,d) were predicted excellently (mean absolute error of around 8 mV). The two membranes prepared at higher concentrations (Figure 8a,c) had slightly higher or, respectively, lower concentrations than the predicted values. To avoid those predictions differing from the actual measurement in the future, adding the new data and recording more underrepresented data will help to improve future predictions.

The comparison between the measured and predicted PWPs for all newly modified membranes is displayed in Figure 9. All predictions were close to the measurements. The standard deviations of the predictions were in the same order of magnitude as the standard deviations of the measurement. The high precision of these predictions showed the extraordinary potential of machine learning approaches for material science studies, in particular for membrane design and improvement. Even with the low amount of data used in this study (only 42 or respectively, 52 data points, i.e., membrane samples), the predictions matched consistently while the time consumption of the predictions was very short (<1 h).

## Conclusions

We showed that small data sets (as they are common in materials science) can be used as training data for machine learning models and enable the reliable prediction of membrane properties. Performance properties, such as pure water permeance and zeta potential of membranes modified with new substances not contained in the training data, were predicted accurately. The predictions were excellent, especially for interpolated values of experimental conditions for the electron-beam-based modification approach, e.g., irradiation dose and concentration. Additionally, the machine learning methods were able to identify the impact of the substance’s chemical structures and process parameters on the resulting membrane properties.

The newly predicted modification substances had chemical properties comparable to those of the training data. Therefore, the predictions were highly reliable. Discrepancies between the predictions and the experimental values were present only at the edges of the trained data space. To avoid this issue in the future, the less well-predicted data generated in this work should be added to the training data. Additionally, more data in these under-represented regions should be added. Therefore, more data from other membrane modifications (e.g., further functional groups, other membrane materials, or additional membrane modification schemes) should be added to improve the predictions of the membrane properties even more systematical. Nevertheless, the predictions were consistently accurate in this study.

In general, using regression models first is recommended when exploring the potential of a small data set because they are easily interpretable and require low computational time. Additionally, using the results predicted from small data sets to identify regions with promising values and interpolate new values is preferred, rather than determining the highest values or predicting values at the edges of the predicted space. The predictions were made in a short computational time with satisfying accuracy. In conclusion, the application of machine learning in membrane modification is a promising tool for accelerating the development of membranes with improved performance and saving time and costs during the development process.

## Supporting Information Available

Characterization of pristine membranes M1 and M2, Figure S1: PEG retention of membrane M1 and M2 at 2 bar transmembrane pressure, Figure S2: scanning electron microscopy images, Table S1: list of membranes prepared, Table S2: feature values, names, CAS number, and structures of the used modification substances, Figure S3: experimental values compared to the training data predicted by the gradient boosting model, and Figure S4: training curves of each training loop for the training and validation data (PDF)

## Abbreviations

DMAEMA: methacrylic acid-2-(dimethylamino)-ethyl ester
DMAPMA: *N*-[3-(dimethylamino)-propyl]-methacrylic amide
DMF: dimethylformamide
GBL: γ-butyrolactone
LMH: L/(m^2^ h)
M1: membrane 1 (prepared from 8 wt % PAN solution)
M2: membrane 2 (prepared from 10 wt % PAN solution)
MAE: mean absolute error
PAN: polyacrylonitrile
PWP: pure water permeance
IEP: isoelectric point
