# Supplementary material for: Design of Modified Polymer Membranes Using Machine Learning
Source: ACS Appl Mater Interfaces. 2024 Apr 11;16(16):20990–1000. doi: 10.1021/acsami.3c18805 (PMC11056926; doi:10.1021/acsami.3c18805)
Supplement: Supplementary file 1 — am3c18805_si_001.pdf [file am3c18805_si_001.pdf]

Supporting Information - Design of modified polymer membranes using machine learning

Sarah Glass[1,2], Martin Schmidt[3], Petra Merten[1], Amira Abdul Latif[3], Kristina Fischer[3], Agnes Schulze[3], Pascal Friederich[2,4,, ‡], Volkan Filiz[1,*,‡]*

1- Institute of Membrane Research, Helmholtz-Zentrum Hereon, Max-Planck-Str. 1, 21502 Geesthacht, Germany

2- Institute of Theoretical Informatics, Karlsruhe Institute of Technology (KIT), Engler-Bunte-Ring 8, 76131 Karlsruhe, Germany

3- Leibniz Institute of Surface Engineering (IOM), Permoserstr. 15, 04318 Leipzig, Germany

4- Institute of Nanotechnology, Karlsruhe Institute of Technology (KIT), Hermann-von-Helmholtz-Platz 1, 76344 Eggenstein-Leopoldshafen, Germany

Corresponding Authors

* Volkan.filiz@hereon.de & pascal.friederich@kit.edu

Characterization of pristine membranes M1 and M2

The pristine membranes were characterized regarding their pure water permeance, molecular weight cut-off (MWCO), surface pore size, surface porosity, and water contact angle (WCA).

Pure water permeance was determined as described before. The MWCO was determined by permeating polyethylene glycol (PEG) solutions (200 mg/l dissolved in water) in dead-end mode cells (at 2 bar transmembrane pressure) through the membranes. To avoid concentration

polarization, the feed solution was stirred at 250 rpm. PEGs of different sizes (98 kDa, 250 kDa, 359 kDa, 504 kDa, 600 kDa) from PSS Polymer Standards Service GmbH (Germany) were used. The MWCO was defined as the smallest molecular weight, which was rejected to more than 90%. The concentrations of PEG in the feed, the retentate and the permeate were measured by gel-permeation chromatography.

Scanning electron microscopy (SEM) was used to determine the surface pore size and porosity. SEM images were recorded on a Merlin SEM (Zeiss, Jena, Germany) at an accelerating voltage of 3 keV using an InLens secondary electron detector. All samples were dried under vacuum at 60 °C for at least 24 h and sputter-coated with a 1 nm thick platinum using a CCU-010 coating device (Safematic, Zizers, Switzerland). The images were analyzed with the software IMS (Imagic Bildverarbeitung AG, Opfikon, Switzerland). The medium pore diameter (pore size) and the area of pores on the surface (surface porosity) were determined. Images at a magnification of 100k were used. Since 1 pixel corresponds to 1.1 nm in size, pores of an area smaller than 3 nm^2 were excluded from the analysis. 3 images of a size of 2.7 μm^2 were analyzed. The mean value and the standard deviation of the three results were used.

The surface roughness R_a and R_q were determined by atomic force microscopy ScanAsyst-Air probe (tip radius ≈ 2 nm, spring constant ≈ 0.4 N/m) in QNM mode from Bruker (Billerica, MA, USA). 3 images of a size of 3 µm x 3 µm were analyzed. The arithmetic average and the standard deviation of the three results were used.

The WCA was measured in sessile drop mode by a Krüss drop shape analyzer (DSA100, Krüss, Hamburg, Germany) and analyzed by the Krüss Advance software (Hamburg, Germany). A droplet (2 µl) of Milli-Q water was placed on the membrane. A picture of the droplet was taken immediately after placing it on the membrane. The average of the contact angles at the left and right sides of the droplet was used to determine the medium contact angle. The medium contact

angle was measured four times on independent samples. The values are given as the mean value ± standard deviation.

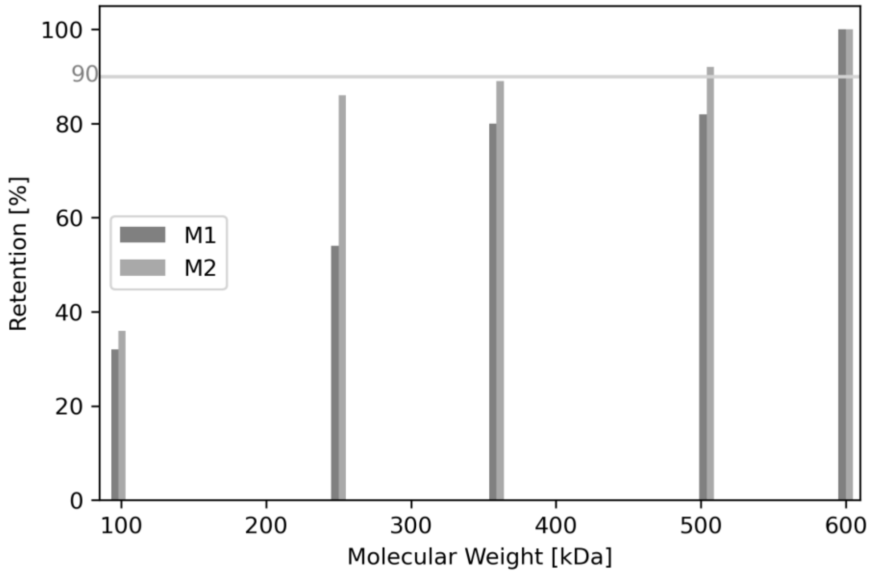

Figure S-1: PEG retention of membrane M1 and M2 at 2 bar transmembrane pressure.

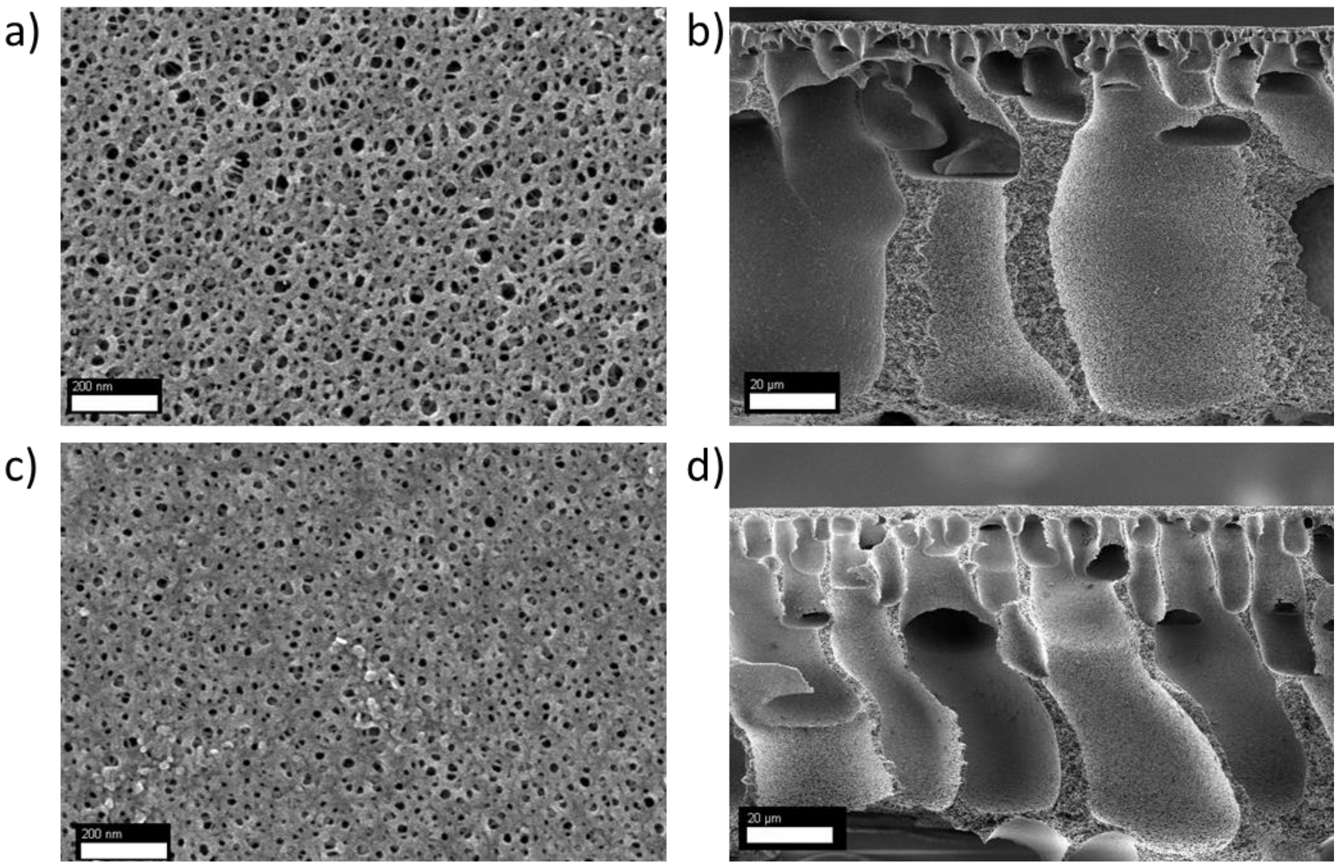

Figure S-2: Scanning Electron Microscopy images of a) the surface of membrane M1, b) the cross-section of membrane M1, c) the surface of membrane M2, and d) the cross-section of membrane M2. Surface images were taken at a magnification of 50k and cross-sectional images were taken at 500 times magnification.

Table S-1: List of membranes prepared. The columns display the zeta potential and the pure water permeance (PWP), if the membrane was characterized using the respective method.

No.	Membrane	Substance	Concentration	Dose [kGy]	Zeta potential	PWP
1	M 2	methacrylamide	5.0	200		x
2	M 2	methacrylamide	10	200	x	x
3	M 2	methacrylamide	1.0	200		x
4	M 2	2-aminoethyl-methacrylamide	5.0	200	x	x
5	M 2	2-aminoethyl-methacrylamide	10.0	200	x	x
6	M 2	2-aminoethyl-methacrylamide	1.0	200		x
7	M 2	[2-(acryloyloxy)ethyl] trimethylammonium	5.0	200	x	x
8	M 2	[2-(acryloyloxy)ethyl] trimethylammonium	20.0	200	x	x
9	M 2	[2-(acryloyloxy)ethyl] trimethylammonium	2.5	200	x	x
10	M 2	[2-(acryloyloxy)ethyl] trimethylammonium	15.0	200	x	x
11	M 2	[2-(acryloyloxy)ethyl] trimethylammonium	10.0	200		x
12	M 2	[2-(acryloyloxy)ethyl] trimethylammonium	1.0	200	x	x
13	M 2	2-(dimethylamino)ethyl acrylate	5.0	200	x	x
14	M 2	2-(dimethylamino)ethyl acrylate	10.0	200	x	x

15	M 2	2-(dimethylamino)ethyl acrylate	1.0	200	x	x
16	M 2	acrylamide	5.0	200		x
17	M 2	acrylamide	10.0	200	x	x
18	M 2	acrylamide	1.0	200		x
19	M 1	(3-methacrylamidopropyl) trimethylammonium	1.0	150	x	x
20	M 1	3-(acryloylaminopropyl) trimethylammonium	5.0	200	x	x
21	M 1	3-(acryloylaminopropyl) trimethylammonium	20.0	200	x	x
22	M 1	3-(acryloylaminopropyl) trimethylammonium	2.5	200	x	x
23	M 1	3-(acryloylaminopropyl) trimethylammonium	1.0	200	x	x
24	M 1	3-(acryloylaminopropyl) trimethylammonium	15.0	200	x	x
25	M 1	3-(acryloylaminopropyl) trimethylammonium	10.0	200	x	x
26	M 1	methacrylamide	5.0	200	x	x
27	M 1	methacrylamide	10.0	200		x
28	M 1	methacrylamide	1.0	200	x	x
29	M 1	2-aminoethyl-methacrylamide	5.0	200	x	x
30	M 1	2-aminoethyl-methacrylamide	1.0	200		x
31	M 1	2-aminoethyl-methacrylamide	10.0	200	x	x
32	M 1	2-trimethyl ammonium ethyl methacrylate	1.0	150	x	x
33	M 1	2-trimethyl ammonium ethyl methacrylate	0.5	200		x

34	M 1	[2-(acryloyloxy)ethyl] trimethylammonium	5.0	200	x	x
35	M 1	[2-(acryloyloxy)ethyl] trimethylammonium	20.0	200	x	x
36	M 1	[2-(acryloyloxy)ethyl] trimethylammonium	2.5	200	x	x
37	M 1	[2-(acryloyloxy)ethyl] trimethylammonium	15.0	200	x	x
38	M 1	[2-(acryloyloxy)ethyl] trimethylammonium	10.0	200	x	x
39	M 1	[2-(acryloyloxy)ethyl] trimethylammonium	1.0	200	x	x
40	M 1	[2-(acryloyloxy)ethyl] trimethylammonium	1.0	150	x	x
41	M 1	[2-(acryloyloxy)ethyl] trimethylammonium	0.5	200	x	x
42	M 1	[2-(acryloyloxy)ethyl] trimethylammonium	0.5	150	x	x
43	M 1	2-(dimethylamino)ethyl acrylate	5.0	200	x	x
44	M 1	2-(dimethylamino)ethyl acrylate	10.0	200	X	x
45	M 1	2-(dimethylamino)ethyl acrylate	1.0	200	x	x
46	M 1	acrylamide	5.0	200	x	x
47	M 1	acrylamide	10.0	200	x	x
48	M 1	acrylamide	1.0	200	x	x
49	M 1	[2-trimethyl ammonium ethyl methacrylate	1.0	150	x	x
50	M 2	(3-acrylamidopropyl) trimethylammonium	0.5	150		x
51	M 2	3-(methacryloylamino) propyl-trimethylammonium	0.5	150		x

52	M 2	[2-(acryloyloxy)ethyl] trimethylammonium	0.5	150	x	x

Table S-2: Feature values, names, CAS number and structures of the used modification substances.

Structure	Name	CAS	pKa	Acryl	Methyl
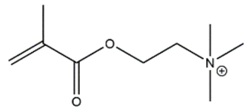	2-trimethyl ammonium ethyl methacrylate	5039-78-1	14.0	1	1
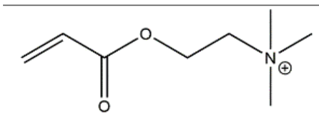	[2-(acryloyloxy)ethyl] trimethylammonium	44992-01-0	14.0	1	0
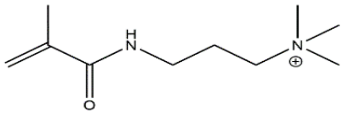	(3-acrylamidopropyl) trimethylammonium	45021-77-0	14.0	0	1
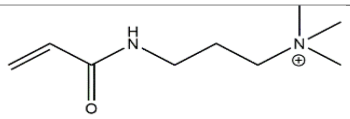	3-(methacryloylamino) propyl-trimethylammonium	45021-77-0	14.0	0	0
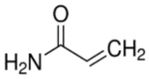	acrylamide	79-06-1	4.2	0	0
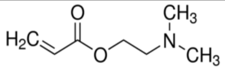	2-(dimethylamino)ethyl acrylate	2439-35-2	8.0	1	0
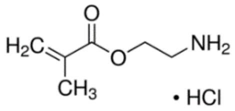	2-aminoethyl-methacrylamide	76259-32-0	8.1	1	1
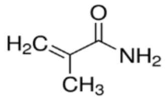	methacrylamide	79-39-0	3.8	0	1
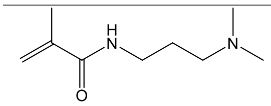	N-[3-(dimethylamino)-propyl]-methacrylic amide (DMAPMA)	5205-93-6	9.4	0	1
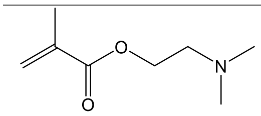	methacrylic acid-2-(dimethylamino)-ethyl ester (DMAEMA)	2867-47-2	8.0	1	1

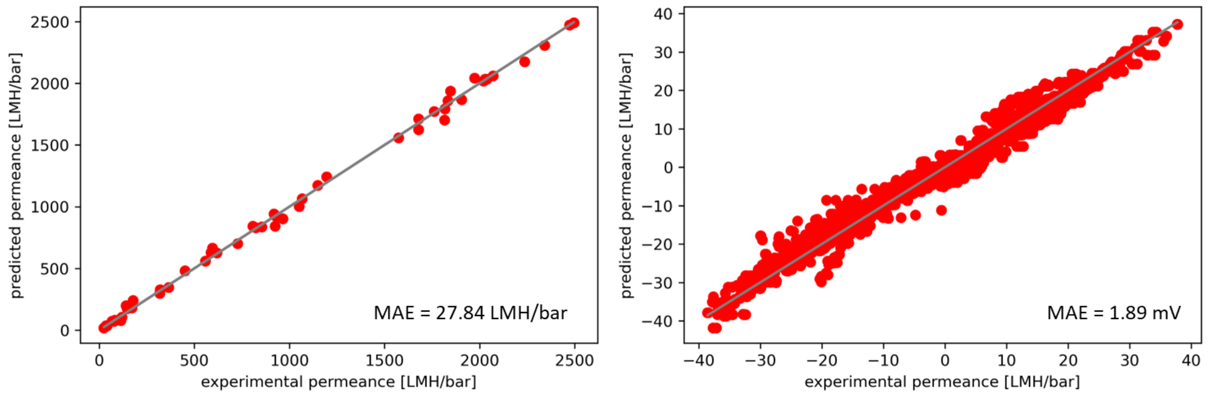

Figure S-3: Experimental values compared to the training data predicted by the gradient boosting model showing the training performance of the leave-one-out cross-validation for (a) PWP and (b) zeta potential.

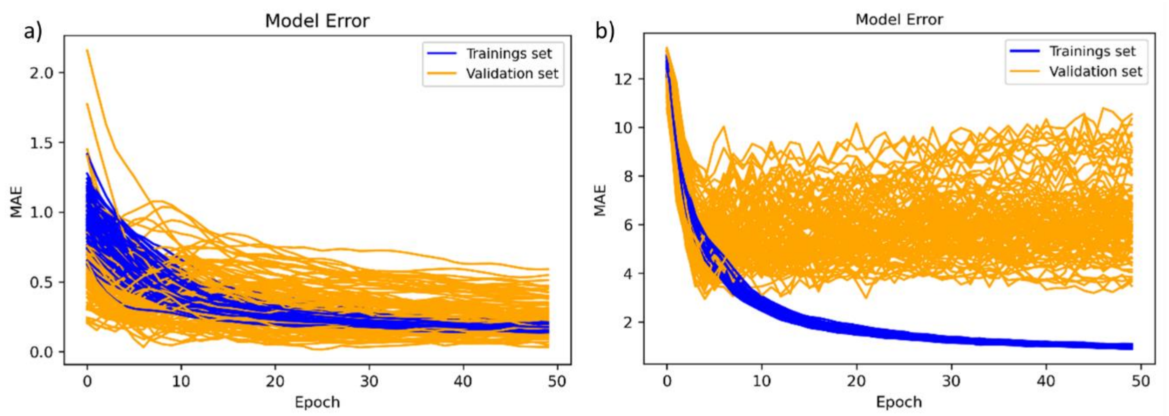

Figure S-4: Training curves of each training loop for the training (blue) and validation data (orange) of a) pure water permeance (PWP) and b) zeta potential.
